# Supplementary material for: A protein secreted by the Salmonella type III secretion system controls needle filament assembly
Source: eLife. 2018 Jul 17;7:e35886. doi: 10.7554/eLife.35886 (PMC6066329; doi:10.7554/eLife.35886)
Supplement: Supplementary file 1. [file elife-35886-supp1.docx]

**Supplementary File 1. Restraints and structural statistics for 20 NMR structures of OrgC**

**_______________________________________________________________**

**Total NOE-derived-distance restraints** 1170

Intraresidue (*i,i*) 583

Sequential (*i, i+1*) 225

Medium Range (2 ≥ | *i – j* | ≤ 4) 189

Long Range (|*i - j*| > 4) 173

**Total dihedral angle restraints** 104

Phi 52

Psi 52

**Root mean square difference from mean structure of helices**

Backbone atoms (N, C^α^, C*’*) (Å) 0.536

All heavy atoms (C, N, O) (Å) 0.960

**Violation analysis**

Max distance violation (Å) 0.40

Max dihedral angle violation (^O^ ) 4.15

**Energies**

Mean generalized Born potential-AMBER energy (kcal mol^-1^) -4479

Mean restraint energy (kcal mol^-1^) 135

**Ramachandran plot** (%)

Most favorable region 83.2

Additionally allowed regions 13.8

Generously allowed regions 2.8

Disallowed regions 0.6

**________________________________________________________________**
