# Supplementary material for: A protein secreted by the Salmonella type III secretion system controls needle filament assembly
Source: eLife. 2018 Jul 17;7:e35886. doi: 10.7554/eLife.35886 (PMC6066329; doi:10.7554/eLife.35886)
Supplement: Supplementary file 2. [file elife-35886-supp2.docx]

**Supplementary File 2. Bacterial strains used in this study**

**Strain Genotype**

SB300 mouse passaged wild type SL1344 strain

SB2942 *orgC* 3xFlag *invC::kan*

SB2943 *orgC* 3xFlag *invJ::kan*

SB2946 *orgC* 3xFlag *spaS*-3xFlag

SB2947 *orgC* 3xFlag spaS^N258A^-3xFlag

SB2326 *∆invJ* *flhD*::Tn*10*

SB2939 *∆invJ∆orgC* *flhD*::Tn*10*

SB3079 *mbp-prgH* *flhD*::*tet*

SB3275 *mbp-prgH* *flhD*::*tet ∆orgC*

SB762 *flhD*::Tn*10*

SB1679 ∆*orgC*

SB2639 ∆*invJ flhD*::Tn*10*

SB2944 ∆*orgC flhD*::Tn*10*

SB3272 *∆invJ invA::kan flhD:*:Tn*10*

SB3273 *∆invJ invG::kan flhD*::Tn*10*

SB3274 *∆invJ ∆prgI flhD*::Tn*10*

SB3289 *∆invJ ∆orgC ∆prgI flhD*::Tn*10*
