# Supplementary material for: A protein secreted by the Salmonella type III secretion system controls needle filament assembly
Source: eLife. 2018 Jul 17;7:e35886. doi: 10.7554/eLife.35886 (PMC6066329; doi:10.7554/eLife.35886)
Supplement: Supplementary file 3. [file elife-35886-supp3.docx]

**Supplementary File 3. Plasmids used in this study**

**Plasmid Description**

pSB3292 *hilA* is under arabinose-inducible promoter in pBAD24 vector

pSB3491 a suicide plasmid to introduce 3xFlag tag at the C-terminus of chromosomal *orgC*

pSB3494 a suicide plasmid to introduce ∆*orgC*

pSB5716 pWSK129-*orgC*

pSB3908 pET15-based expression vector for N-terminally 6xHis-tagged OrgC

pSB5713 pET15-based expression vector for N-terminally 6xHis-and 3xFlag-tagged OrgC

pSB3931 *orgC* was cloned in pT25 plasmid for the bacterial two-hybrid assay

pSB5851 pT18-*orgC*

pSB5871 pWSK129-*orgC-3xF*

pSB5718 pWSK129*-∆N21orgC-3xF*

pSB3915 pWSK129*-∆N21orgC*

pSB3904 pWSK129-*mbp*

pSB3905 pWSK129-*mbp-orgC*

pSB3935 pT18-*prgI∆C5*

pSB5719 pWSK129-*orgC*(∆134-136)

pSB5720 pWSK129-*orgC*(∆137-140)
